# The impact of K-beauty social media influencers, sponsorship, and product exposure on consumer acceptance of new products

**DOI:** 10.1186/s40691-020-00239-0

**Published:** 2021-04-05

**Authors:** Lei Wang, Jin Hwa Lee

**Affiliations:** 1 Doctoral Candidate, Dept. of Clothing and Textiles, Pusan National University, 316-1, 2, Busandaehak-ro 63beon-gil, Geumjeong-gu, Busan, South Korea; 2 Professor, Dept. of Clothing and Textiles, Pusan National University, 306, 2, Busandaehak-ro 63beon-gil, Geumjeong-gu, Busan, South Korea

**Keywords:** K-beauty, Social media influencers (SMIs), Sponsorship display status, Product exposure method, Factorial design, Acceptance intention toward new products

## Abstract

This research identified the different ways in which K-beauty social media influencers (SMIs) impact consumers’ tendencies to accept new products by closely investigating their sponsorship displays and product exposure methods. We conducted an experiment to examine how influencers’ posts affect Chinese millennial consumers’ acceptance of new products. We used a 2 (influencer type: celebrity versus general public) × 2 (sponsorship display status: display versus no display) × 2 (product exposure method: exposure versus no exposure) factorial design. The findings reveal that the social media posts that made consumers most likely to accept a new product were created by a general public influencer and did not include a display of any sponsorship affiliation with the product. Additionally, there was a significant interplay between the influencer type, sponsorship display status, and product exposure method regarding consumers’ acceptance intention toward a new product. A consumer was most likely to accept and purchase a new product when three factors (general public influencer, sponsorship displayed, and product exposed) were combined. Based on the findings, we draw important implications and present marketing strategies for companies in the beauty industry that use SMI marketing.

## Introduction

As social media becomes more widespread, consumers are acquiring information from diverse channels (Rauniar et al. 2014). Businesses strategically utilize various types of digital content and platforms to build and develop relationships with these consumers (Hanna et al. 2011), as well as marketing strategies that rely on influential individuals, or “influencers,” who live in the limelight (Freberg et al. 2011). From a marketing perspective, an influencer is a type of information communicator who affects the perceptions, attitudes, and behaviors of the information acceptor, and an influencer can make a considerable impression on the marketing target (Cho & Cho 2013).

Extensive exposure to advertisements on social media has increased user fatigue, which, in turn, has led users to avoid advertisements altogether (Kelly et al. 2010). A survey on the causes of social media fatigue syndrome reported that the main culprit is the increased number of bold and aggressive marketing and promotional posts by companies (Bright et al. 2015). In digital marketing research, the influencer marketing strategy is drawing attention as an effective marketing method to increase consumer participation (Dhanesh & Duthler 2019). Furthermore, as increasing amount of information is delivered via social media, the acquisition of reliable information has emerged as a key issue for consumers. Additionally, the role of influencers as opinion leaders who can significantly affect consumer perspectives is growing (Van den Bulte & Joshi 2007). Influencers who are members of the general public can exert enormous influence on social media (which is easily accessible through mobile devices), making them equivalent to celebrities.

The global market has witnessed a dramatic increase in exports of Korean cosmetics. In a short period, K-beauty has successfully attracted worldwide attention (Lee & Lee 2018) and, along with Hallyu (the Korean Wave), K-beauty is on the rise. Simultaneously, K-beauty influencers on social media are becoming more important because they act as easily accessible resources from which consumers can gather information (Wang & Lee 2019). Many Chinese consumers who are affected by the Korean Wave are highly interested in K-beauty influencers (Kang et al. 2020). Chinese consumers access K-beauty content from social media influencers (SMIs) on sites such as Weibo (a social media platform that is similar to Twitter/Instagram). They also review and utilize K-beauty content in an interactive manner. As influencers become responsible for a larger proportion of sales, marketing that uses K-beauty SMIs is occurring and rapidly evolving. Additionally, many brands are expanding their marketing channels to include K-beauty SMIs. In this context, it is necessary to examine the role of K-beauty SMIs in the Chinese cosmetic industry (Wang & Lee 2019).

Most Chinese consumers will look for and carefully read relevant product posts written by SMIs before buying cosmetics (Kang et al. 2020). Cosmetics companies and related professionals often hire beauty influencers (a celebrity or a member of the general public can be a K-beauty influencers) to post about new products on social media (Korea Trade-Investment Promotion Agency (KOTRA 2017), especially Weibo posts that feature sponsorship displays and product exposure. Therefore, when it comes to K-beauty SMI marketing, it is important to consider multiple variables, such as the type of K-beauty influencer, the use of sponsorship display status, and product exposure methods. Prior research has shown that these three components have a significant impact on consumer response; therefore, scholars and marketing professionals may benefit from a deeper understanding of these components. First, it is important to choose the right influencer. Traditional celebrity influencers and general public influencers offer unique benefits for marketers. While a celebrity influencer may reach a larger consumer market for brands, general public influencers can deliver, for example, higher returns on investments (ROIs) (Trivedi 2018). Second, the sponsorship display status in posts has a potential impact on the posts’ effectiveness. Influencers may directly or indirectly promote new products or a businesses’ brand appeal on social media. By using “sponsorship” tags in posts, marketers give consumers a sense of the messages’ commercial intent, and which helps consumers identify ads (Friestad & Wright 1994). Third, the manner in which products are presented is also important in influencer marketing. According to product placement research (Russell 2002), consumers are more likely to accept a product if it appears organically in a post. However, if a product is placed in a considerably prominent position and does not seem to be related to the scene, consumers react with negative perceptions and feel it is an inelegant advertisement (Cowley & Barron 2008). In short, influencer type, sponsorship display status, and product exposure are three variables generally present in influencer posts. However, the interaction among these variables has not been the focus of previous research. Therefore, our research investigated whether Chinese millennials are influenced by marketing elements like the K-beauty SMI type, sponsorship display status, and method of product exposure.

Beauty SMIs’ enormous influence and disseminating power in the cosmetics industry prompt consumers to accept and purchase new products; the importance of this trend cannot be overemphasized (Schouten et al. 2019). Studies that explore whether SMIs’ K-beauty posts actually lead to Chinese millennial consumers intending to purchase these beauty items will be important with regard to future influencer marketing. Although studies about social media and influencer attributes have been conducted, few empirical studies have examined consumers’ behavioral intentions as they relate to K-beauty SMIs (Choi & Lee 2019). This study examines how posts and information disseminated by K-beauty SMIs impact Chinese millennial consumers’ acceptance of new products, thus contributing to the literature on corporate strategy, marketing strategy, and beauty influencer marketing.

In modern times, public consumption behavior tends to revolve around social media rather than mass media (Aksoy et al. 2013). In light of this situation, this study builds on existing research to establish and discuss the various types of K-beauty SMIs. In addition, with the rise of influencer marketing, there are several commercial cases of this type of marketing. In this regard, we examine whether displaying “sponsorship” (which here refers to the relationship, if any, the influencer has with the company that sells the product) in K-beauty SMIs’ posts impacts the effects of marketing. Furthermore, this study explores how the effectiveness of influencer marketing can be maximized. To this end, we investigate whether exposing products (which here refers to the product appearing in the post) via K-beauty SMIs offering recommendations to their Chinese millennial online audiences is effective in terms of information delivery and new product promotion. Finally, we explore whether the interaction of these marketing variables is effective.

As described above, this study classifies the types of K-beauty SMIs and empirically explores the effects of product exposure channels and displaying sponsorship status, as well as the interaction between the two, on customer intentions. We aim to identify how these factors influence Chinese millennial consumers’ acceptance intentions regarding new products. Furthermore, we investigate the influence of SMIs and propose practical ideas for effective marketing and communication strategies for firms involved in the K-beauty industry and SMI marketing.

## Literature review

### The possibility of K-beauty entering the Chinese market

Today, with the development of social media in China, consumers’ interest in beauty is increasing, and the cosmetics market in China is thus growing rapidly. Interest in the brand K-beauty is growing in particular in the nation. In 2019, the value of Korean cosmetics exported to China was $24.37 billion, and the import share was 25.2%, ranking second (Japan ranked first with 25.5%) (Han 2019). From 2012–2018, the average annual growth rate of the Chinese cosmetics market was 6.52%, and the growth rate of Korean cosmetics imports in China was 14% in 2019 (KOTRA 2020). When young female consumers in China purchase K-beauty products, most purchases occur alongside social media likes on Weibo after referring to an influencer’s recommendations or reviews. Skin-care products account for more than 52% of the total sales of K-beauty products, as they are considered the most promising items due to their reputation for reliability (KOTRA 2020). In addition, there is a trend in which new products that are highly popular in Korea, such as BB cream and the CC cushion, attract significant interest in China (KOTRA 2019).

As the Chinese beauty market evolves, the demand for social media beauty content increases. With the creation of various consumption trends and channels in China, the opportunity to enter the consumption market has expanded. Consequently, the segmentation of consumers and the platform-construction of unique consumption channels are accelerating with the growth of revolutionized media such as influencers and power blogs. Additionally, the purchasing power of Chinese millennials has grown remarkably. In addition, other factors such as the increase of China’s middle class, more direct overseas trade, growing demand for premium consumer goods, and the expansion of the import market centered on the beauty, health, and high-end food sectors have allowed for K-beauty products to enter the Chinese market continuously (KOTRA 2019). Therefore, when K-beauty companies enter the Chinese market by utilizing new distribution channels, such as SMI marketing and O2O platforms, they need an appropriate marketing strategy. Thus, in this study, we will examine how K-beauty influencers affect Chinese consumers on Chinese social media and present the findings as important basic data for companies that will use K-beauty SMI marketing strategies in the future.

### K-beauty social media influencers

The term influencer was coined by adding the suffix “er” (to indicate a person) to “influence” (“to have an impact on”) and refers to a person who exerts influence (De Veirman et al. 2017; Lou & Yuan 2019; Oh 2019). With the emergence of influencers, who have a strong impact on many consumers who are social media users, the way companies communicate with consumers has also changed (Freberg et al. 2011). Influencers embody fun and empathy, and companies collaborate with influencers to introduce and promote their brands. Rather than promoting new products and services directly to consumers, companies indirectly promote these items through influencers (Djafarova & Rushworth 2017). The act of promoting a company’s products and services through influencers’ content via various social media channels is called influencer marketing (Mun & Kim 2020). In this digital age, the importance of social media, which serves as a space for users to share information, experiences, and opinions, has increased. An increasing number of people are being influenced by social media, and 47% of millennials state that social media has a direct effect on their purchasing behavior (Opus 2016). This is in strong contrast to other age groups, among which only 19% of people responded similarly. Many companies are aware of the importance of social media and attempt to utilize it to promote corporate brands or new products (Opus 2016).

Influencers create content on various subjects–beauty, travel, food, broadcasting, and video games. In particular, beauty items appeared as the subjects of content in the early days of social media, and this content remains popular with female consumers. Influencers in this field strive to form lasting relationships with consumers by creating and sharing content on makeup skills and cosmetic product reviews. People who conduct these activities are called beauty influencers, beauty creators, or one-person media (Kim 2017). In this context, the beauty influencers who provide information on Korean cosmetic products on social media are called K-beauty SMIs (Wang & Lee 2019).

As digital technology and marketization rapidly develop simultaneously, consumers have an increased desire for new products (Nylén & Holmström 2015). K-beauty companies introduce new products to this market to satisfy the needs of consumers and foster intentions to accept these new products. For example, the Korean cosmetic company Amorepacific’s hair-care brand “Ryo” has brought classic Korean aesthetics and hairstyles to the Chinese market. Korean celebrity Park Shin-hye was invited to create posts and share them through social media, such as Weibo in China, which recorded approximately 3.18 million people seeing these posts. Sales increased by 6.7% compared to that in the same period in the previous year (Wang & Lee 2019).

Weibo has more official accounts of K-beauty SMIs than any other site, satisfying the desires of Chinese consumers for various information related to K-beauty (Kim & Ahn 2012). K-beauty is also a derivative product of Hallyu, and Chinese consumers’ interests in K-beauty has expanded to the cosmetics, hairstyles, makeup, skin care, and nails mentioned by K-beauty SMIs (An 2013). K-beauty influencers provide information and share opinions on social media platforms such as Weibo regarding new products on the market. They expand and enhance their leverage by using their professional knowledge and displaying their integrity. Thus, they impact consumers’ acceptance intentions (Lim et al. 2017). Additionally, according to Lee (2015), influencers deliver information to their followers, recommend new products, and continuously build and maintain relationships with consumers. All these actions induce consumers to develop positive acceptance intentions. The rise of K-beauty SMIs is considered to be the leading factor in the globalization of K-beauty because they have elevated beauty to entertainment. Their content has rapidly expanded and been reproduced worldwide via social media (Jung 2020). Additionally, traditional retailers are facing a decline in on-site shoppers as consumers spend more time shopping online (Baek et al. 2020). The K-beauty industry, which is suffering due to the spread of COVID-19, is actively targeting the Chinese online market. It aims to take advantage of the online consumer culture that emerged from the COVID-19 pandemic while capturing the millennial generation, which is an emerging major consumer in the Chinese beauty market (Lee 2020). In this context, this study investigates the effects of K-beauty influencers on consumers’ acceptance intentions toward new products. The study also presents new data to firms that plan to adopt the K-beauty influencer strategy.

### Types of influencers

Existing studies categorize the types of influencers based on certain classification criteria. Depending on their activities, the types are “beauty influencer,” “fashion influencer,” and “food influencer.” Influencers can be further divided into “creators” (who create their own content) and “models” (who promote existing items) (KOTRA 2017). Influencers can also be categorized based on the number of followers they have. Mega influencers have more than 1 million followers, macro influencers have between 100,000 and 1 million followers, micro influencers have between 1000 and 100,000 followers, and nano influencers (also called potential influencers) have fewer than 1000 followers (Gilpark 2018).

As social media becomes more popular and widespread, the importance of influencers becomes more evident. General public influencers are becoming as successful and informative as celebrities, offering fashion advice and introducing beauty products through social media (Jin & Muqaddam 2019; Lin et al. 2018). Celebrity influencers can use their own attributes, such as physical appeal, to gain followers’ trust and achieve the desired marketing effect (Shin & Han, 2019). A study by Atkin and Block (1983) also showed that consumers prefer to listen to celebrities. General public influencers, on the other hand, tend to be more relatable and connect more closely to consumers by having an effect similar to that of a warm-hearted neighbor or a trusted friend with whom consumers can create a sense of intimacy (Berryman & Kavka 2017; Son & Kim 2017). Thus, both general public influencers and celebrities are important variables in terms of consumer responses (Berryman & Kavka 2017; Kolo & Haumer 2018; Schouten et al. 2019). Previous research has shown that selecting the right influencer can be challenging as different types of influencers produce different advertising results. Although different types of influencers play a pivotal role in marketing communications (Tsang & Zhou 2005), little research has been conducted on the differences in consumer responses to celebrity and general public influencer content. The key question that must be answered is how consumers react to celebrity and general public influencers in the K-beauty social media environment. Therefore, this study compares these two types of influencers to explore consumers’ reactions when the same product is promoted.

In some studies that have investigated influencer marketing, influencers were classified as information providers. SMIs who engage in the cosmetic industry are called beauty creators, and influencers in the social media environment are called information providers (Choi & Behm-Morawitz 2017; Dekavalla 2019). Different types of beauty influencers have different effects on consumers. General public influencers have more influence on young consumers’ purchasing behaviors and attitudes about brands attitudes compared to celebrity influencers (Schouten et al. 2019; Trivedi & Sama 2019). However, findings (Trivedi 2018) suggest that attractive celebrity influencers have a greater influence on consumers’ reactions in the fashion and lifestyle industries than generalist influencers. In addition, this study reported that general public influencers in the cosmetics field had a greater influence on consumers than celebrities did (Choi & Behm-Morawitz 2017; Dekavalla 2019). Therefore, the first hypothesis of this study is as follows:

### H1. Consumers’ acceptance intentions regarding new products will differ depending on the influencer type

#### Sponsorship display status

Extant empirical studies (Forrest & Cao 2010; Thorson & Rodgers 2006) have identified two types of beauty influencers who recommend products. One type creates content by purchasing and personally using the product, and the other type creates content with material and financial support (e.g., being sponsored for sharing a product). The latter type indicates to their audience that the content and product are sponsored. This is referred to as a “sponsorship display” (Park 2015). Therefore, in this study, the term “sponsorship display” refers to cases in which beauty influencers have received financial and material support from a cosmetics company and relay to their audience that the content was sponsored.

According to the Federal Trade Commission (FTC), influencers must provide a clear sponsorship statement declaring a relationship with a brand. However, it is difficult for consumers to perceive sponsored influencer posts as advertisements (FTC 2013). Sponsorship displays, in turn, help consumers identify advertisements (Friestad & Wright 1994). Raising awareness of advertising by flagging sponsored messages can increase consumer trust in influencers, which can have a positive impact on consumers’ responses (Boerman 2020). In influencer posts, sponsorship disclosure does not impair purchase intent (Müller et al. 2018). Simultaneously, other studies have shown that social media users perceive posts as advertisements if they perceive these posts as persuasion knowledge. This results in a negative impact on consumer behavior (Friestad & Wright 1994; Kim & Kim 2020). This can be explained by the persuasion knowledge model (PKM: Friestad & Wright 1994). Previous studies related to influencer marketing have shown that sponsorship displays can have different effects on different research subjects. Therefore, it becomes important to study the impact of sponsorship display statuses in K-beauty SMIs’ posts.

Previous studies have found that audiences generally respond negatively to sponsorship or rewards (Lee 2007; Ryu 2004). Lee (2007) studied the differences in effects as they related to the display of an article-type advertisement. When identical messages were presented, the messages that clearly displayed that the advertisement was, in fact, an advertisement had lower promotional potential compared to the cases in which the advertisement’s nature was obscured or not displayed at all. Ryu (2004) studied consumer responses to rewarded versus organic word-of-mouth information provided online and found that respondents responded less favorably to the rewarded information. In particular, audiences inferred that the firm’s intention regarding providing online word-of-mouth information was a corporate marketing activity (Bataineh 2015; Lin & Lu 2010). Similarly, it is necessary to investigate how audiences perceive sponsored content because the general trend is that influencer marketing is becoming more popular on social media. Thus, we hypothesize the following:

### H2. Consumers’ acceptance intentions regarding new products will differ depending on the sponsorship display status.

#### Product exposure method

Babin and Carder (1996), who studied product placement (PPL) in film, classified PPL into two types: “on-set placement” and “creative placement.” In on-set placement, the product stands out and is referred to or used by the actor(s), while creative placement means the product is simply placed in the background. Another classification was developed by Gupta and Lord (1998), who used the concept of prominence to divide the types into “prominent placement” and “subtle placement.” In this study, their operational definition was almost the same as that of onset-creative placement. The Korean researchers Kim (2004) and Kim and Bong (2013) divided PPL into media and exposure types and reclassified PPL by exposure based on the following four scenarios: direct exposure of a product or service, logo exposure, direct references to a product, and a product or service portrayed as background material.

Product exposure has many advantages, such as raising brand awareness among viewers (Cowley & Barron 2008), creating positive connections to memory and choice (Law & Braun 2000), and having a positive impact on brands (Russell 2002). However, prominent product displays can activate persuasive knowledge, which makes viewers aware of persuasive intentions and thus leads to negative reactions (Cowley & Barron 2008). These results are closely related to influencer posts where product exposure positively impacts viewers, however, the negative effects of product exposure should not be ignored. Therefore, this study explores the impact of product exposure methods in SMI posts on K-beauty consumers.

Product exposure can have a positive impact on purchasing behavior, but its potential negative impact cannot be disregarded. Previous studies have noted that the extent of the exposure matters. In other words, customers’ negative responses to content increase as the exposure increases (Homer 2009; van Reijmersdal et al. 2009). Based on prior studies’ findings that consumer responses differ according to product exposure methods, this study distinguished between products that were and were not exposed on social media. The former refers to a post that actually shows the product, whereas the latter refers to a post where the product is only mentioned. It was predicted that consumer responses would differ depending on whether the product was exposed on social media. Therefore, the following hypothesis is proposed:

### H3. Consumers’ acceptance intentions regarding new products will differ depending on the product exposure method

#### Interaction effects

We have previously discussed the main roles of influencer type, sponsorship display status, and methods of product exposure. These elements often manifest in diverse combinations, and these different combinations can have different effects. Therefore, it is important to understand how these combinations affect consumers. Previous research has not provided much insight into the different combinations of these elements. Simultaneously, a complete understanding of the interactions between these elements can make influencers’ posts more accurate and effective. Therefore, this study clarifies whether these different combinations of elements generate positive interactions and which combinations effectively influence Chinese millennials to accept new products.

### Interaction between influencer type and sponsorship display

When discussing influencer marketing, it is difficult to ignore how influencers post content that may be sponsored by a brand or company. Although SMIs are required to clearly label sponsored content, research shows that they sometimes fail to do so (Liljander et al. 2015). As influencer marketing activities increase, the commercial utilization of influencers also increases rapidly (Childers et al. 2018). However, the side effects of these activities also emerge (Soh 2012). One influencer testified about receiving a request from a firm not to indicate to social media users that the product was sponsored to maximize the advertising effect (Cho & Cho 2013). According to a study by Kim and Lee (2017), a friend’s recommendation is more likely to convince a consumer to purchase a product, even if a celebrity’s content lacks a sponsorship disclosure. Sparkman and Richard (1982) studied changes in the persuasive effects of advertisements based on consumer speculations on the number of fees received by certain celebrities. The study reported that consumers were less influenced by advertisements when they speculated that the celebrities were paid high fees. The results of these studies suggest consumers doubt the sincerity of a celebrity influencer’s intentions when they believe the influencer is only recommending a certain product for a material reward. In this context, the following hypothesis is proposed:

### H4. Consumers’ acceptance intentions regarding new products will be affected by the interaction between the influencer type and the sponsorship display status

#### Interaction between influencer type and product exposure method

Marketers believe social media is a great channel to promote their products to their target audience. Finding the right influencer and optimal method to showcase a product is the best way to maximize brand visibility (Trendhero 2020). Russell and Rasolofoarison (2017) argued that product placement can have a positive impact on purchase intentions and that this impact depends on the extent to which consumers like the celebrity (or influencer). A recent study by Schouten et al. (2019) examined the effect of influencer type on advertising effectiveness and found that a general public influencer had a greater influence on consumer behavioral intentions than did direct exposure to the product. This finding indicates that the interaction between influencer type and product exposure has a strong effect on consumer behavioral intentions. Therefore, we posit the following hypothesis:

### H5. Consumers’ acceptance intentions regarding new products will be affected by the interaction between the influencer type and the product exposure method

#### Interaction between sponsorship display status and product exposure method

Next, we will discuss the two-way interaction of sponsorship display status and product exposure method. According to previous research, when sponsorship is displayed or when the product is exposed, both can have an impact on consumers. However, if both of these interactions reveal the business intentions behind the social media post (Friestad & Wright 1994; Kim & Kim 2020; van Reijmersdal et al. 2009), how this interaction impacts consumers’ new product acceptance intentions is worth exploring.

Chu et al. (2016) found that people prefer product exposures where advertising is either non-existent or not prominent. Similarly, Ewers (2017) concluded that when there is no exposure of the product, it is more advantageous with regard to the lack of a sponsorship display as a sponsorship display can show the advertising context to the audience. Based on these findings, it is expected that the display of sponsorship and the product exposure method interact with each other in the social media environment to affect consumers’ acceptance of new products. Therefore, the following hypothesis is proposed:

### H6. Consumers’ acceptance intentions regarding new products will be affected by the interaction between the sponsorship display status and the product exposure method

#### Interaction between influencer type, sponsorship display status, and product exposure method

Finally, a thorough understanding of the three-way interaction between these elements will allow companies that use influencer marketing to combine them more effectively. This study explored the interaction effects that may occur between all three independent variables. Dekker and van Reijmersdal (2010) reported that disclosing to consumers that the content is an advertisement or is sponsored can reduce the effect of product exposure when the influencer is perceived as dishonest. Based on existing findings in the literature, it was predicted that each two-way interaction (influencer type and sponsorship display status, influencer type and product exposure method, and sponsorship display status and product exposure method) would affect consumers’ acceptance intentions toward a new product. It was also predicted that the influencer type, sponsorship display status, and product exposure method would impact consumer responses via three-way interactions. Based on the above discussion, this study hypothesizes the following:

H7. Consumers’ acceptance intentions regarding new products will be affected by the interaction between the influencer type, sponsorship display status, and product exposure method

## Methods

### Study design

**Fig. 1:**
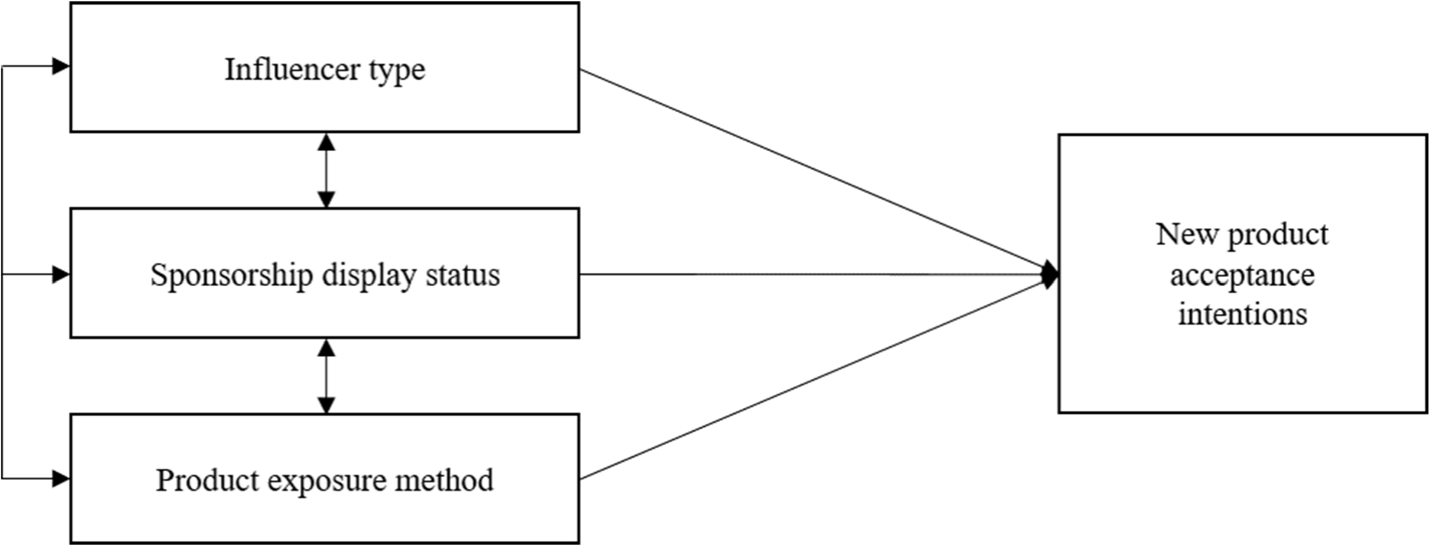
Research model

This study aimed to verify the effect of K-beauty SMIs and sponsorship and product exposure on consumers’ new product acceptance intentions, as shown in Fig. 1. An online survey was conducted using a 2 × 2 × 2 between-group factor design, including influencer type (celebrity versus general public), sponsorship display status (display versus no display), and product exposure method (exposure versus no exposure). This design led to eight different experimental stimuli and eight experimental groups, which are displayed in Appendix 1. The online questionnaire comprised stimuli and questionnaire items. Only one stimulus was randomly selected and presented in the questionnaire.

### Stimulus material and manipulation checks

**Fig. 2:**
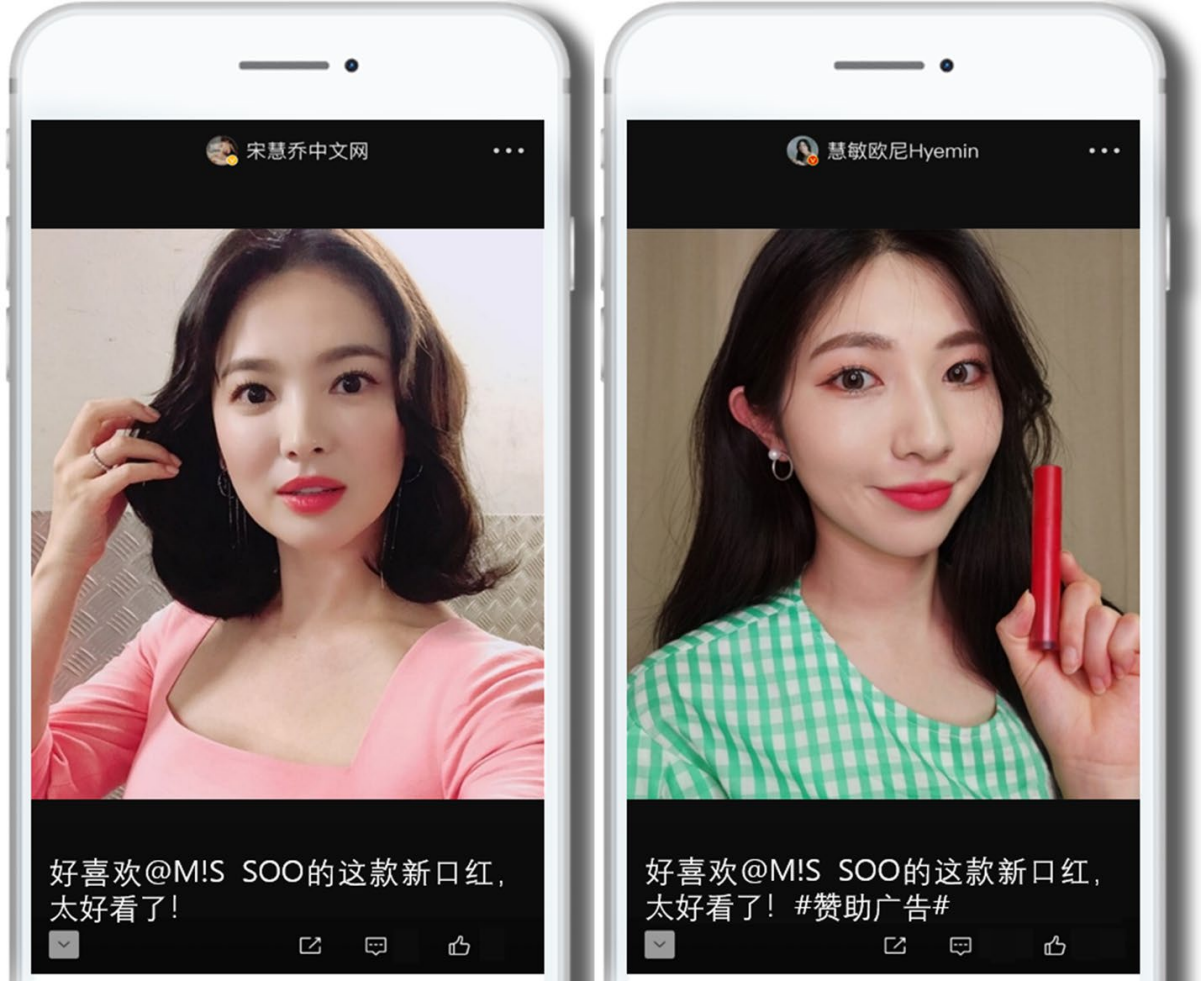
Fictitious Weibo post from a celebrity influencer with no sponsorship display and no product exposure (left); Fictitious Weibo from a general public influencer with sponsorship display and product exposure (right)

Weibo is the leading social media platform in China, and it allows SMIs to create photos or short videos independently (e.g., without complicated video production processes). SMIs can use these produced photos and videos to appeal to followers, and they can effectively communicate with followers with functions such as hashtags on social media. In this study, for a more realistic effect, eight fictitious Weibo posts were created to produce stimuli (see Fig. 2 for two example images of the stimuli). They were created in Chinese based on typical influencer posts and message compositions. A preliminary survey was conducted with a convenience sample of 80 Chinese female undergraduate and graduate students (in their 20 s and 30 s), all of whom live in China, to perform a manipulation check of the stimuli.

### Fictitious Korean brand “M!S SOO”

Regarding the product brand, if a real brand is selected, the experimental results may be biased due to the predispositions of the participants (Lee & Lee 2007). Therefore, a fictitious Korean brand called “M!S SOO” was created for use in this study to control for predisposed brand attitudes. The preliminary survey was designed in part to determine and test whether this fictitious Korean brand “M!S SOO” was recognized. The survey began by providing respondents with an explanatory statement that “M!S SOO” is a fictitious Korean cosmetics brand. Subsequently, the survey asked the respondents to select “Yes” or “No” to the question “I already knew about the Korean cosmetics brand ‘M!S SOO’ even before participating in this survey.” All respondents answered that they did not know the brand “M!S SOO.” Thus, we confirmed that the fictitious brand was created successfully.

### Influencer type

Based on a Weibo survey (as of March 2020) that measured users’ favorite Korean Wave celebrities with at least 438,000 Weibo users, Song Hye-kyo earned 117,000 votes and ranked first among 10 candidates (Vote-Weibo 2020). Therefore, Song Hye-kyo was selected as a celebrity influencer for this study. In addition, a general public influencer named Hyemin was selected based on the number of followers, frequency of activities (frequent posts), and content composition. Hyemin has 1.41 million followers on Weibo, similar to the celebrity influencer Song Hye-kyo (1.2 million), and the cosmetics in their posts are mainly K-beauty products (as of March 2020). Song Hye-kyo uploads 2–3 posts per week and offers various cosmetic activities promotion content, cosmetic recommendations, and updates about film and television works and daily life. However, Hyemin uploads a bit more frequently (2–5 posts per week) and offers content that mainly focuses on cosmetics recommendations and daily life (as of March 2020). The personal photos presented in the influencers’ Weibo accounts were used for stimulus development, and they were manipulated under two influencer type conditions (celebrity/general public). In this regard, the images of the influencer (celebrity/general public) were shown, and the ratio between the figure size and the total screen was composed similarly in the two conditions (see Appendix 2 for original images of the two types of influencers used in the experiment). Therefore, we judged the conditions to be suitable for use in the experiment. A preliminary survey was conducted to determine whether the subject was properly aware of the type of influencer. After exposing the two conditions (of influencer type) randomly to the respondents in the preliminary survey, the participants responded to four items (“Many people know the name of this influencer,” “Movies/dramas/programs with this influencer are popular,” “This influencer is a celebrity,” and “This influencer is famous,” Cronbach’s α = 0.987). These were supplemented or modified from Kim and Whang (2019) and Morrin et al. (2006), using a 5-point Likert scale that ranged from 1 (strongly disagree) to 5 (strongly agree). As a result of analyzing the response data with a paired sample t-test, it was judged that the two conditions showed significant differences (*t* = 21.09, *p* < 0.001) and that the manipulation of influencer type was appropriate (*M*_*celebrity*_ = 4.89, *M*_*general public*_ = 1.78).

### Sponsorship display status

As the sponsorship display must be shown in the post caption, a stimulus’s caption must be formulated like a real Weibo post. Each caption comprised the brand name, a positive message about the product, and the influencer’s opinion of the proposed product. This caption mimicked the format of an existing Weibo post. Consequently, the following caption was created: “@M!S SOO I really like the new lipstick. It’s so pretty!” In addition, the hashtag “#sponsorship ad#” was added to the caption under the sponsorship display conditions. The preliminary survey also allowed us to determine whether the sponsorship display status (display/no display) of each stimulus was properly recognized. The two sponsorship display status conditions were presented at random to the preliminary survey respondents. Based on previous studies (Tutaj & van Reijmersdal 2012; van Reijmersdal et al. 2016), they responded via a 5-point Likert scale (1 = “strongly disagree” to 5 = “strongly agree”) to the four items (“The influencer indicated that the post was sponsored,” “I think the influencer was compensated by the brand for creating the post,” “The post contained a #sponsored ad# hashtag,” and “I think this post has become an advertisement,” Cronbach’s α = 0.986) that were modified/supplemented. An analysis of the response data with a paired sample t-test revealed that the two conditions showed significant differences (*t* = 6.61, *p* < 0.001), indicating that the manipulation of sponsorship display status was successful (*M*_*display*_ = 4.39, *M*_*no display*_ = 2.36).

### Product exposure method

Regarding product selection, according to a report released by the data center CBNData in November 2019, the largest number of Chinese consumer reviews of beauty content comprised reviews of color cosmetics. Therefore, a red lipstick, which scored highest in the color cosmetics interest ranking, was selected as the product to be used in this study (Data Center 2019). In this study, the research was conducted by dividing the posts into cases in which the product was exposed on social media and cases in which the product was not exposed. This was manipulated under the two conditions for the product exposure method (exposure/no exposure). To appropriately manipulate each stimulus for the product exposure conditions, a photo of a hand holding red lipstick without the brand name or logo exposed was edited using Photoshop and added to the experimental image. Through the preliminary survey, it was confirmed that the respondents properly recognized the product exposure method of the stimulus. The two conditions for the product exposure method were displayed randomly to the preliminary survey respondents. Based on the research of Babin and Carder (1996) and Panda (2003), the respondents used a 5-point Likert scale (1 = “strongly disagree” to 5 = “strongly agree”) to respond to the four modified/supplemented items (“The product mentioned by the influencer could not be seen in the post (reverse-coded),” “The influencer was holding the product in her hand,” “The influencer showed the product she mentioned,” and “The product the influencer was referring to was visible in the post,” Cronbach’s α = 0.972). An analysis of the response data with a paired sample t-test revealed that the two conditions showed significant differences (*t* = 21.66, *p* < 0.001), indicating that the manipulation of the product exposure method was successful (*M*_*exposure*_ = 4.68, *M*_*no exposure*_ = 1.59).

### Measurement tools

In the main experiment, a measurement tool based on a scale developed in a previous study was modified and used according to this study’s purpose. To measure consumers’ new product acceptance intentions, four items (“I am favorable toward this new product,” “I think it’s wise to buy this new product,” “I am generally satisfied with this new product,” and “I have an intention to use this new product,” Cronbach's α = 0.920) were adapted from DeLone and McLean (1992) and Wang and Lee (2019). A 5-point Likert scale was used (1 = “strongly disagree” to 5 = “strongly agree”) to measure responses. In addition, to gain insight into the sample’s characteristics, the survey included four questions on demographic characteristics that were measured with a nominal scale.

### Data collection and analysis

This study aimed to empirically verify the impacts of influencer type, sponsorship display status, and product exposure method of K-beauty SMIs on the new product acceptance intentions of Chinese millennial consumers. To this end, the respondents were female consumers from the millennial generation (born between the early 1980s [1980–1982] and the early 2000s [2000–2004]) who at the time were active on Chinese social media and used Weibo. The main survey was conducted from April 24–26, 2020, based on a random sample of 816 female millennial consumers in China. For data collection, we requested to Wen Juanxing (a professional online survey service contractor) and developed a questionnaire website. The website URL for the questionnaire was sent and returned via a Chinese social networking site (WeChat).

The respondents were randomly assigned one of eight questionnaires. Only one stimulus was presented randomly in every questionnaire. The respondents who saw a given stimulus were first asked to answer the manipulation confirmation question (influencer type, whether sponsorship was displayed, product exposure method) to check that the stimulus was properly recognized. The questionnaire responses from 16 participants who did not respond to the exact conditions of the manipulated stimuli were excluded. Therefore, 800 results (98.04%) were used for the final analysis (see Appendix 1), which comprised only the respondents who accurately responded to the stimuli. Following the manipulation test questions, the respondents answered questions about their new product acceptance intentions for each stimulus. Finally, they answered questions on demographic characteristics. The data collected in this study were analyzed with SPSS 25.0.

The data on the sample’s demographic characteristics indicated that 32.3% of the respondents were 25–29 years old, 26.8% were between 20–24 years old, 26.2% were 30–34 years old, and 14.7% were 35–39 years old. Regarding marital status, there was an even distribution (50.1% single and 49.9% married). For academic background, 39.6% of the respondents were university graduates, 26.4% were university students, 20.3% were graduate students or higher, and 13.8% were high school graduates or lower. Regarding current occupation, 43.0% were office workers, 28.0% were students, 11.0% were public officials, 8.9% were housewives, and 2.8% were self-employed.

### Pretest

Chi-square analyses and one-way ANOVA tests were performed to check for randomization and group equivalence. First, for the participants in each of the eight experimental groups, chi-square analyses were conducted for demographic characteristics. Across the experimental groups, the comparison of the demographic characteristics of the respondents in each group is presented in Table 1. The results of the chi-square analyses showed no significant difference among the eight experimental groups with respect to age (*χ*^2^ = 8.266, *df* = 21, *p* = 0.994), marital status (*χ*^2^ = 0.195, *df* = 7, *p* = 0.998), academic background (*χ*^2^ = 7.297, *df* = 21, *p* = 0.997), and current occupation (*χ*^2^ = 19.322, *df* = 35, *p* = 0.985).

**Table 1:** Demographic characteristics of participants in eight experimental groups

	Group 1 n = 100	Group 2 n = 100	Group 3 n = 100	Group 4 n = 100	Group 5 n = 100	Group 6 n = 100	Group 7 n = 100	Group 8 n = 100	Total N = 800
Age									
20–24	26	28	27	30	21	26	28	28	214
25–29	32	31	26	29	35	34	36	35	258
30–34	30	26	30	25	27	25	22	25	210
35–39	12	15	17	16	17	15	14	12	118
*Χ*^2^ = 8.266, *df* = 21, *p* = 0.994									
Marital status									
Single	49	49	51	50	50	51	51	50	401
Married	51	51	49	50	50	49	49	50	399
*Χ*^2^ = 0.195, *df* = 7, *p* = 0.998									
Academic background									
High school graduate or lower	13	14	15	12	13	13	17	13	110
University student	26	24	30	28	25	23	26	29	211
University graduate	40	38	38	42	44	38	38	39	317
Graduate student or higher	21	24	17	18	18	26	19	19	162
*Χ*^2^ = 7.297, *df* = 21, *p* = 0.997									
Current occupation									
Student	30	27	28	29	29	26	29	26	224
Office worker	37	39	49	47	47	50	37	38	344
Public official	10	12	11	9	10	8	14	14	88
Housewife	12	12	7	6	6	7	10	11	71
Self-worker	2	3	1	3	3	3	4	3	22
Not employed/ others	9	7	4	6	5	6	6	8	51
*Χ*^2^ = 19.322, *df* = 35, *p* = .985									

Frequency

Next, to confirm randomization and group equivalence of the participants, one-way ANOVA tests were performed to assess the participants’ personal attitudes toward the SMI and Korean cosmetics pre-experiment. The results of the one-way ANOVA tests showed no significant difference among the eight experimental groups with regards to pre-experimental personal attitudes toward the SMI (measured with one item on a 5-point semantic differential scale: “You feel this influencer is 1 (unlikable) to 5 (likable)”), *F*_7,792_ = 0.49, *p* = 0.841. Further, there were no significant differences regarding pre-experimental personal attitudes toward Korean cosmetics (assessed with one item on a 5-point semantic differential scale: “Korean cosmetics are 1 (bad) to 5 (good)”), *F*_7,792_ = 0.44, *p* = 0.877. These results suggested that the random assignment was conducted correctly, and group equivalence was ensured (Jin & Ryu 2019; Stubb & Colliander 2019).

## Results

### Factor analysis and reliability analysis of measurement variables

Table 2 shows the results of the exploratory factor analysis and Cronbach’s α values for the new product acceptance intention scale with the K-beauty SMIs, sponsorship display, and product exposure method. The results were derived in four independent dimensions by conducting a factor analysis by Varimax rotation using principal component analysis (Cumulative percent of variance explained = 91.629, KMO = 0.875, Bartlett’s test = 19,004.263, *p* = 0.000). The factor placement of each question was 0.800 or higher, verifying the construct validity of the factors. Cronbach’s α values for the four scales were all above 0.900, confirming that the reliability of the measurement was at a relatively acceptable level.

**Table 2:** Results of exploratory factor analysis

Constructs	Factor loading	Cronbach’s *alpha*
Sponsorship display status		0.984
The influencer indicated that the post was sponsored	0.978	
I think the influencer was compensated by the brand for creating the post	0.977	
The post contained a #sponsored ad# hashtag	0.976	
I think this post has become an advertisement	0.975	
Eigenvalue = 3.818 Percent of variance explained = 23.861		
*Influencer type*		0.983
Many people know the name of this influencer	0.975	
Movies/dramas/programs with this influencer are popular	0.974	
This influencer is a celebrity	0.969	
This influencer is famous	0.968	
Eigenvalue = 3.806 Percent of variance explained = 23.787		
Product exposure method		0.981
The product mentioned by the influencer could not be seen in the post. ^a^	0.978	
The influencer was holding the product in her hand	0.974	
The influencer showed the product she mentioned	0.971	
The product the influencer was referring to was visible in the post	0.969	
Eigenvalue = 3.794 Percent of variance explained = 23.714		
New product acceptance intentions		0.920
I am favorable toward this new product	0.925	
I think it’s wise to buy this new product	0.906	
I am generally satisfied with this new product	0.881	
I have an intention to use this new product	0.868	
Eigenvalue = 3.243 Percent of variance explained = 20.267		

^a^ Reverse-coded item

Next, (a) the influencer types were divided into two groups based on the median split, which was the method used by Baek and Hwang (2018). The median was obtained from the responses to the four questions after the variable transformed. With reference to the median of 3.00, values that were the same or higher were classified as the celebrity influencer group (50%), and those that were lower than the median were classified as the general public influencer group (50%). (b) With reference to the median number of 3.00 for sponsorship display status, values that were the same or higher than the median were classified as the display group (50%) and values that were lower than the median were classified as the no display group (50%). (c) With reference to the median number of 3.13 for the product exposure method, values that were the same or higher than the median were classified as the exposure group (50%), and those that were lower than the median were classified as the no exposure group (50%).

### Three-way ANOVA

To address the research problem, three categorical variables—influencer type (celebrity versus general public), sponsorship display status (display versus no display), and product exposure method (exposure versus no exposure)—were set as independent variables. The dependent variable was the consumers’ new product acceptance intentions. For the possible main and interaction effects of the independent variables, a three-way ANOVA was performed to determine which effects were significant. The three-way ANOVA results (Table 3) showed two significant main effects and four significant interaction effects. A more detailed look at these effects is provided in the following subsections.

**Table 3:** Results of three-way ANOVA

Variable	Sum of squares	*df*	Mean Square	*F*
Influencer type (A)	17.11	1	17.11	292.97^***^
Sponsorship display status (B)	0.69	1	0.69	11.82^**^
Product exposure method (C)	0.09	1	0.09	1.55
A × B	343.88	1	343.88	5887.68^***^
A × C	94.19	1	94.19	1612.64^***^
B × C	0.36	1	0.36	6.19^*^
A × B × C	4.50	1	4.50	77.05^***^
Error	46.26	792	0.06	
Total	12745.38	800		

^*^ *p* < 0.05, ^**^ *p* < 0.01, ^***^ *p* < 0.001

### Main effects

#### New product acceptance intentions according to influencer type

First, the main effect of influencer type on consumers’ new product acceptance intentions was significant, as shown in Table 3 (*F*_1,792_ = 292.97, *p* < 0.001). In the analysis of variance test, testing for interaction effects can only identify whether the overall population mean of several groups is the same. Specifically, to check whether there is a difference in means between specific populations, it is necessary to use a multiple comparison method. The simple main effect test, one of the multiple comparison methods, checks to determine whether there is a difference in means between different groups of factors while controlling the conditions of one factor (Gang 2014). A simple main effect analysis was conducted to test the differences in new product acceptance intentions according to the type of influencer. The results confirmed that the general public influencer (*M*_*general public*_ = 4.06) had a stronger impact on consumers’ new product acceptance intentions than did the celebrity influencer (*M*_*celebrity*_ = 3.77) (*F*_1,792_ = 292.97, *p* < 0.001). Therefore, the results support hypothesis 1.

### New product acceptance intentions according to sponsorship display status

Table 3 shows the significance of the main effect of sponsorship display status on consumers’ new product acceptance intentions (*F*_1,792_ = 11.82, *p* < 0.01). A simple main effect analysis was conducted to test the differences in new product acceptance intentions depending on sponsorship display status. The no display group (i.e., the group that was not exposed to a display of sponsorship) (*M*_*no display*_ = 3.94) had higher rates of new product acceptance intentions than did the display of sponsorship group (*M*_*display*_ = 3.88) (*F*_1,792_ = 11.82, *p* < 0.01). Therefore, the results support hypothesis 2.

### New product acceptance intentions according to the product exposure method

The main effect of the product exposure method on consumers’ new product acceptance intentions was not significant, as shown in Table 3 (*F*_1,792_ = 1.55, *p* > 0.05). Therefore, hypothesis 3 can be rejected.

### Two-way interaction effects

#### New product acceptance intentions according to influencer type and sponsorship display status

Table 3 shows the interaction effect of influencer type and sponsorship display status on consumers’ new product acceptance intentions (*F*_1,792_ = 5887.68, *p* < 0.001). By examining the interaction effect with a simple main effect analysis, regarding sponsorship display, the general public influencer (*M*_*general public*_ = 4.68) had a more significant impact on consumers’ new product acceptance intentions than did the celebrity influencer (*M*_*celebrity*_ = 3.08) (*F*_1,792_ = 4403.69, *p* < 0.001) (Table 4). On the other hand, in the case of no sponsorship display, the results indicate that the celebrity influencer (*M*_*celebrity*_ = 4.45) had a more positive impact on consumers’ new product acceptance intentions than did the general public influencer (*M*_*general public*_ = 3.43) (*F*_1,792_ = 1776.96, *p* < 0.001). Therefore, the results support hypothesis 4.

**Table 4:** Simple main effect for two-way interaction of influencer type × sponsorship display status

Dependent Variable	Sponsorship display status	Influencer type	*M*	*F*
New product acceptance intentions	Display	Celebrity	3.08	4403.69^***^
		General public	4.68	
	No display	Celebrity	4.45	1776.96^***^
		General public	3.43	

^***^ *p* < 0.001

### New product acceptance intentions according to influencer type and product exposure method

Table 3 shows the interaction effect of influencer type and product exposure method on consumers’ new product acceptance intentions (*F*_1,792_ = 1612.64, *p* < 0.001). By examining the interaction effect with a simple main effect analysis, the results indicate the general public influencer (*M*_*general public*_ = 4.39) had a greater impact on consumers’ product acceptance intentions than did the celebrity influencer (*M*_*celebrity*_ = 3.41) (*F*_1,792_ = 1640.16, *p* < 0.001) in the case of product exposure (Table 5). On the other hand, when the product was not exposed to the participant, the celebrity influencer (*M*_*celebrity*_ = 4.12) had a more positive impact on consumers’ acceptance intentions of the new product than did the general public influencer (*M*_*general public*_ = 3.73) (*F*_1,792_ = 265.45, *p* < 0.001). Therefore, the results support hypothesis 5.

**Table 5:** Simple main effect for the two-way interaction of influencer type × product exposure method

Dependent Variable	Product exposure method	Influencer type	*M*	*F*
New product acceptance intentions	Exposure	Celebrity	3.41	1640.16^***^
		General public	4.39	
	No exposure	Celebrity	4.12	265.45^***^
		General public	3.73	

^***^ *p* < 0.001

### New product acceptance intentions according to sponsorship display status and product exposure method

Table 3 shows the interaction effect of the product exposure method and sponsorship display status on consumers’ new product acceptance intentions (*F*_1,792_ = 6.19, *p* < 0.05). A simple main effect analysis was conducted to investigate the detailed differences. The results of this analysis are shown in Table 6, which indicates that when the product was exposed, the no sponsorship display condition (*M*_*no display*_ = 3.91) has a more positive impact on consumers’ new product acceptance intentions than did the sponsorship display condition (*M*_*display*_ = 3.85) (*F*_1,792_ = 6.96, *p* < 0.01). On the other hand, when the product was not exposed, there is no difference in new product acceptance intentions between the two groups (sponsorship display versus no sponsorship display) (*F*_1,792_ = 0.77, *p* > 0.05). Therefore, the results partially support hypothesis 6.

**Table 6:** Simple main effect for the two-way interaction of sponsorship display status × product exposure method

Dependent Variable	Product exposure method	Sponsorship display status	*M*	*F*
New product acceptance intentions	Exposure	Display	3.85	6.96^**^
		No display	3.91	
	No exposure	Display	3.95	0.77
		No display	3.93	

^**^ *p* < 0.01

### Three-way interaction effect

Table 3 shows the interaction effect of influencer type, sponsorship display status, and product exposure method on consumers’ new product acceptance intentions (*F*_1,792_ = 77.05, *p* < 0.001). A simple interaction effect analysis was performed (Table 7), and the results indicate that, in the case of product exposure, there was a significant interaction effect between the influencer type and sponsorship display status (*F*_1,792_ = 2308.82, *p* < 0.001). Additionally, in the case of no product exposure, there was still a significant interaction effect between the other two factors (*F*_1,792_ = 3655.84, *p* < 0.001).

**Table 7:** Simple interaction effect for the three-way interaction

	Product exposure method	Sum of Squares	*df*	Mean Square	*F*
Influencer type × sponsorship display status	Exposure	134.85	1	134.85	2308.82^***^
	No exposure	213.53	1	213.53	3655.84^***^

^***^ *p* < 0.001

**Fig. 3:**
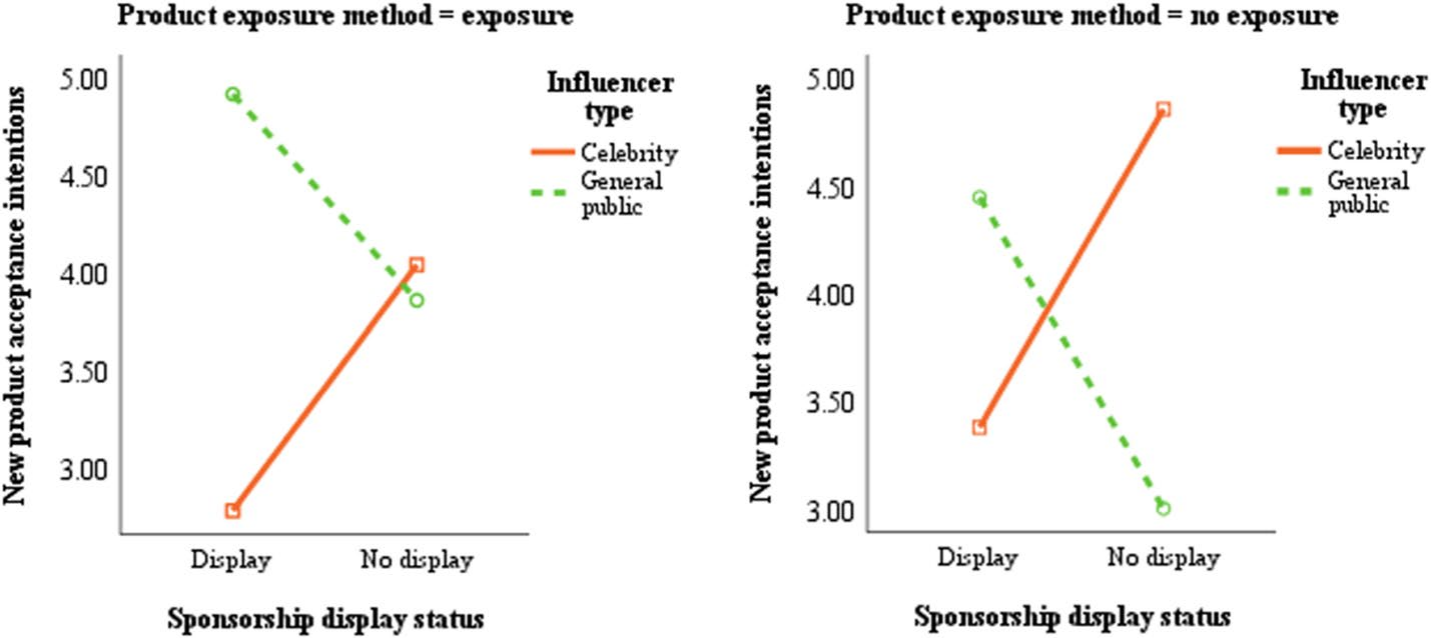
Three-way interaction of influencer type × sponsorship display status × product exposure method

A simple main effect analysis was conducted to investigate the detailed differences. These results are displayed in Table 8 and Fig. 3 and indicate that when the consumer was exposed to a product and the sponsorship was displayed, the general public influencer (*M*_*general public*_ = 4.92) had a greater impact on consumers’ new product acceptance intentions in comparison to the celebrity influencer (*M*_*celebrity*_ = 2.78) (*F*_1,792_ = 3920.49, *p* < 0.001). On the other hand, when the consumer was exposed to a product and the content lacked a sponsorship display, the celebrity influencer (*M*_*celebrity*_ = 4.04) had a more positive impact on consumers’ new product acceptance intentions than did the general public influencer (*M*_*general public*_ = 3.86) (*F*_1,792_ = 28.51, *p* < 0.001). However, when the product was not exposed to the consumer and the sponsorship was displayed, the general public influencer (*M*_*general public*_ = 4.45) had a more positive effect on consumers’ new product acceptance intentions in comparison to the celebrity influencer (*M*_*celebrity*_ = 3.38) (*F*_1,792_ = 975.55, *p* < 0.001). Conversely, when the product was not exposed to the consumer and the content lacked a display of sponsorship, the celebrity influencer (*M*_*celebrity*_ = 4.86) had a greater impact on consumers’ new product acceptance intentions than did the general public influencer (*M*_*general public*_ = 3.00) (*F*_1,792_ = 2945.78, *p* < 0.001). Therefore, the results support hypothesis 7.

**Table 8:** Simple main effect for the three-way interaction

Dependent variable	Product exposure method	Sponsorship display status	Influencer type	*M*	*F*
New product acceptance intentions	Exposure	Display	Celebrity	2.78	3920.49^***^
			General public	4.92	
		No display	Celebrity	4.04	28.51^***^
			General public	3.86	
	No exposure	Display	Celebrity	3.38	975.55^***^
			General public	4.45	
		No display	Celebrity	4.86	2945.78^***^
			General public	3.00	

^***^ *p* < 0.001

## Conclusions

In this study, we focused on K-beauty SMIs to examine how influencer types, sponsorship displays, and product exposure methods affect Chinese millennial consumers’ new product acceptance intentions. The findings of this study are significant in that they are relevant to the rapidly growing and diversifying Chinese beauty market, and the study is relevant because it conducts empirical research on millennial consumers who are active on social media.

This study examined the differences in consumers’ tendencies to accept products featured in K-beauty SMIs’ social media content, and in looking at the type of influencer, sponsorship display status, and method of product exposure, it made three major findings. First, the results indicate that consumers’ new product acceptance intentions were relatively high when the influencer was a member of the general public and when there was no display of sponsorship. These results are similar to those found in previous research that showed that generalist influencers had a more positive impact on younger consumers than did celebrity influencers (Schouten et al. 2019; Trivedi & Sama 2019). Posts with sponsorship disclosures generated fewer likes and comments than did posts without sponsorship disclosures (Hendriks et al. 2020). In addition, there was no significant difference according to the product exposure method. The results showed that even exposure to the product had no effect on consumer reaction. Similar to the previous research, the exposure of the product did not positively persuade consumers (Boerman & van Reijmersdal 2016). Second, there were two-way interaction effects. Specifically, in the case of the general public influencer when the sponsorship was indicated, and in the case of the celebrity influencer when the sponsorship was not indicated, consumers were more likely to accept the new product. Our results showed that consumers’ acceptance intentions regarding new products were affected by the interaction between the influencer type and the sponsorship display status. This is consistent with previous research showing that consumers’ attitudes toward the information source (e.g., the influencer) can become negative if they see a sponsorship disclosure (Boerman et al. 2015). Moreover, in the case of the general public influencer when the product was exposed, and in the case of a celebrity influencer when the product was not exposed, there was a more positive effect on consumers’ new product acceptance intentions. Our first main finding suggests that the product exposure method does not have a positive impact on consumers, but a positive two-way interaction is produced when it works in conjunction with influencer type. This gives us reason to believe in the importance of influencer type. Previous research has also found that product placements by generalist influencers tend to be effective in gaining likes and eliciting more positive brand attitudes if the generalist influencers have similar followings as those of traditional celebrities (Hayes & Carr 2015). Consumers react differently to PPL on influencers’ accounts, depending on the presence or absence of influencers. Consumers react negatively to influencers’ posts when they do not appear with the products they endorse (Jin & Muqaddam 2019; Lin et al. 2018; Lou et al. 2019). Additionally, when the product was exposed, the results indicated that consumers’ new product acceptance intentions were higher when the sponsorship was not displayed. This result is consistent with the previous research. d’Astous and Seguin (1999) pointed out how the more obtrusive a PPL in television sponsorship is, the more negative consumers’ evaluations that result from it. The third major finding is that there was a three-way interaction effect; specifically, when the product was exposed, sponsorship was displayed, and the influencer was a member of the general public, this resulted in the strongest positive effect on consumers’ new product acceptance intentions. On the other hand, when the product was not exposed and when there was no display of sponsorship, the results indicate the celebrity influencer had a greater influence on consumers’ new product acceptance intentions. Our findings provide new support for influencer marketing. We also found there is no significant difference in sponsored display status when the product is not exposed. However, a positive three-way interaction is produced when the two work in conjunction with the proper influencer type, again demonstrating the important role of influencer type.

## Implications

This study has academic significance as it analyzed consumer behaviors regarding new K-beauty products. Specifically, it approached the subject by focusing on the acceptance behaviors of Chinese millennial consumers engaging with K-beauty SMIs. There have been several studies conducted on K-beauty, but these studies have rarely focused on SMIs (Fedorenko 2017; Kim et al. 2013; Yoo & Jin 2015; Yu et al. 2015). Other studies have discussed social media characteristics and attitudes toward advertising content (Ashley & Tuten 2014; Goh et al. 2013; Lee et al. 2018; Knoll 2015). However, with K-beauty SMIs, an increasing number of consumers are demonstrating that they use K-beauty products by reviewing the influencer-created content (Wang & Lee 2019). In this regard, this study identified various factors that may influence how Chinese consumers accept new K-beauty products, thereby broadening the scope of research on K-beauty SMIs. In particular, the findings of this study demonstrated the importance of influencers by showing that Chinese consumers have more positive acceptance intentions regarding new products when they are introduced to the product by a general public influencer. In a previous study that investigated influencers by type, it was reported that consumers trust the content provided by general public influencers more than they trust the content provided by celebrity influencers, which is in agreement with the findings of this study (Kim et al. 2019). In addition, there was a significant interaction effect between the influencer type and product exposure method regarding the influence on Chinese consumers' new product acceptance intentions. Specifically, the findings highlighted that consumers had more positive new product acceptance intentions when a general public influencer engaged in product exposure and when a celebrity influencer did not engage in product exposure. By focusing on the types of influencers that have been somewhat overlooked in previous research, this study confirmed that the effects of influencers should be considered in combination with specific product exposure methods, thus providing significant academic implications.

Another significant implication of this study is that it identified the importance of the presence of (or lack thereof) sponsorship displays in influencer marketing. Other studies have shown that an influencer’s effect differs by sponsorship display status. In this study, the findings illustrate that even when consumers were provided with the same content, the content that displayed its sponsorship had less of an advertising effect in comparison to content with either subtle or no sponsorship displays (Lee 2007). The results of this study confirm that in posts by a K-beauty influencer, consumers’ new product acceptance intentions are stronger when sponsorship is not displayed. However, when there is a significant interaction effect between the influencer type and sponsorship display status on consumers’ new product acceptance intentions, for general public influencers, this study confirmed that consumers’ acceptance of the product was higher when sponsorship was displayed.

Another academic implication of this research is that it demonstrated that in beauty influencer marketing, depending on the relationship between the influencer type, sponsorship display status, and product exposure method, the impact on consumers’ new product acceptance intentions can differ. This finding expands the scope of research on new product acceptance. Existing research on beauty influencers has mainly focused on the effects of attitudes, evaluations, and purchase intentions regarding products. However, this study confirmed that K-beauty SMIs can influence consumers’ new product acceptance intention. Consumers had the greatest positive new product acceptance intentions with the following combination: a general public influencer, a sponsorship display, and product exposure.

Regarding a practical implication of this study, firms that plan to utilize influencer marketing should consider employing general public influencers who command relatively lower fees rather than celebrities who have higher fees (Choi & Cheong 2017). Based on this study’s finding that the general public influencer had a more positive effect on consumers’ new product acceptance intentions, it is fair to conclude that the influence of the general public influencer is higher. This finding reaffirms that employing celebrity influencers on social media platforms should be re-evaluated (Kim & Whang 2019).

This study also offers a practical solution regarding the conflict between materially rewarded posts and the reduced effectiveness of content that displays such sponsorship (Lee 2014). The Federal Trade Commission recommends that any economic interests that exist between an advertiser and a recommender should be publicly disclosed (FTC 2013; Kim & Whang 2019). This study found that when a general public influencer was employed for product marketing, the new product acceptance intention was higher in the case of sponsorship displays. Based on these results, firms that implement influencer marketing should hire general public influencers and display the sponsorship instead of simply hiring a celebrity influencer and hiding the sponsorship.

In this study, we identified the differences in consumers’ new product acceptance intentions by influencer type, sponsorship display status, and product exposure method. In line with the recent increase in the importance of influencer marketing, we presented an influencer marketing strategy and discussed how such a strategy can vary by the type of influencer. This study and its results provide theoretical data and practical guidelines for firms and related parties engaged in the beauty industry to effectively utilize beauty influencers, thus facilitating the establishment of a marketing strategy. Another significant implication of this study is that it identified that the influencer type, sponsorship display status, and product exposure methods have both main and interaction effects on the new product acceptance intentions of Chinese millennial consumers.

## Limitations and future research

The limitations of this study are as follows. First, we used only one model and photo for each influencer type and for each product exposure status. Different models and products can vary significantly and are thus likely to generate different consumer reactions. While we used specific models (Song Hye-kyo and Hyemin) and a specific product photo (one of red lipstick), a combination of more diverse models and different types of products (e.g. creams, foundations) can increase the credibility of and lead to more diversified research results. Future research should thus explore variations of influencers and products. Second, as this research was exclusively concerned with Chinese millennial consumers, there is a limit to generalizing the results of the research. Studying different generations may reveal different consumer acceptance intentions. In addition, the consumption capacity of Generation Z (those born 1997–2012) is growing rapidly, and the growth rate is much higher than that of other age groups (Data Center 2019). Therefore, future work should include comparative studies using millennials and members of Generation Z as participants. We propose studying this topic further by broadening the scope to encompass multiple countries and regions and include comparative studies using millennials and members of Generation Z as participants. Despite these limitations, our findings are expected to add to the literature on SMI marketing related to the beauty industry and should be useful to practitioners as well.

## Figures and Tables

**Fig. 4:**
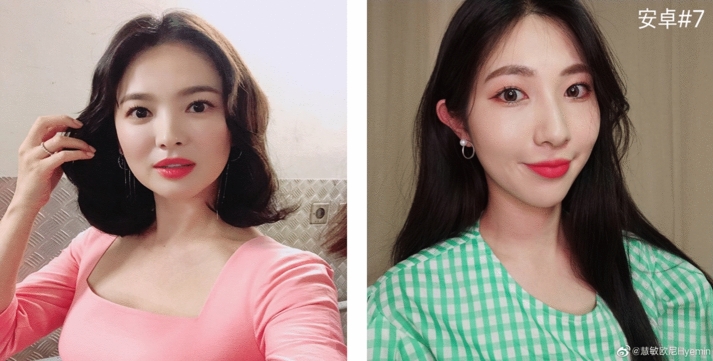
Original images of the two types of influencers used in the experiment. Celebrity influencer type—Song Hye-kyo (left) (The source of the image: a post presented on Song Hye-kyo’s Weibo account on August 29, 2018, https://m.weibo.cn/status/4278457582202732); General public influencer type—Hyemin (right) (The source of the image: a post presented on Hyemin’s Weibo account on June 11, 2019, https://m.weibo.cn/status/4382042596968596#&gid=1&pid=2)

## Data Availability

The datasets used and/ or analyzed during the current study are available from the corresponding author on reasonable request.

## Appendix 1

See Table 9.

**Table 9:** Description of the eight experimental stimuli/eight experimental groups and the sample size per stimulus/per group

Stimulus number	Experimental group	Influencer type	Sponsorship display status	Product exposure method	Sample size
1	Group 1	Celebrity	Display	Exposure	100
2	Group 2	Celebrity	Display	No exposure	100
3	Group 3	Celebrity	No display	Exposure	100
4	Group 4	Celebrity	No display	No exposure	100
5	Group 5	General public	Display	Exposure	100
6	Group 6	General public	Display	No exposure	100
7	Group 7	General public	No display	Exposure	100
8	Group 8	General public	No display	No exposure	100

## Appendix 2

See Fig. 4.

## Abbreviations

FTC: Federal Trade Commission
KOTRA: Korea Trade-Investment Promotion Agency
ROIs: Returns on investment
SMIs: Social media influencers
PPL: Product placement
